# Hetero-antagonism of avibactam and sulbactam with cefiderocol in carbapenem-resistant *Acinetobacter* spp.

**DOI:** 10.1128/spectrum.00930-24

**Published:** 2024-08-20

**Authors:** Olivia Wong, Vyanka Mezcord, Christina Lopez, German Matias Traglia, Fernando Pasteran, Marisel R. Tuttobene, Alejandra Corso, Marcelo E. Tolmasky, Robert A. Bonomo, María Soledad Ramirez

**Affiliations:** 1 Center for Applied Biotechnology Studies, Department of Biological Science, College of Natural Sciences and Mathematics, California State University Fullerton, Fullerton, California, USA; 2 Unidad de Genómica y Bioinformática, Departamento de Ciencias Biológicas, CENUR Litoral Norte, Universidad de la República, Salto, Uruguay; 3 Laboratorio Nacional/Regional de Referencia en Antimicrobianos, Instituto Nacional de Enfermedades Infecciosas, ANLIS Dr. Carlos G. Malbrán, Buenos Aires, Argentina; 4 Área Biología Molecular, Facultad de Ciencias Bioquímicas y Farmacéuticas, Universidad Nacional de Rosario, Rosario, Argentina; 5 Instituto de Biología Molecular y Celular de Rosario (IBR, CONICET-UNR), Rosario, Argentina; 6 Research Service and GRECC, Louis Stokes Cleveland Department of Veterans Affairs Medical Center, Cleveland, Ohio, USA; 7 Departments of Medicine, Pharmacology, Molecular Biology and Microbiology, Biochemistry, Proteomics and Bioinformatics, Louis Stokes Veterans Affairs Medical Center, Cleveland, Ohio, USA; 8 CWRU-Cleveland VAMC Center for Antimicrobial Resistance and Epidemiology (Case VA CARES), Cleveland, Ohio, USA; University of Manitoba, Winnipeg, Manitoba, USA

**Keywords:** *Acinetobacter*, antagonism, cefiderocol, antimicrobial susceptibility testing (AST), avibactam, sulbactam, carbapenem-resistance

## Abstract

Cefiderocol, a siderophore-cephalosporine conjugate antibiotic, shows promise as a therapeutic option for carbapenem-resistant (CR) *Acinetobacter* infections. While resistance has already been reported in *A. baumannii*, combination therapies with avibactam or sulbactam reduce MICs of cefiderocol, extending its efficacy. However, careful consideration is necessary when using these combinations. In our experiments, exposure of *A. baumannii* and *A. lwoffii* to cefiderocol and sulbactam or avibactam led to the selection of cefiderocol-resistant strains. Three of those were subjected to whole genome sequencing and transcriptomic analysis. The strains all possessed synonymous and non-synonymous substitutions and short deletions. The most significant mutations affected efflux pumps, transcriptional regulators, and iron homeostasis genes. Transcriptomics showed significant alterations in expression levels of outer membrane proteins, iron homeostasis, and β-lactamases, suggesting adaptive responses to selective pressure. This study underscores the importance of carefully assessing drug synergies, as they may inadvertently foster the selection of resistant variants and complicate the management of CR *Acinetobacter* infections.

**IMPORTANCE:**

The emergence of carbapenem-resistant *Acinetobacter* strains as a serious global health threat underscores the urgent need for effective treatment options. Although few drugs show promise against CR *Acinetobacter* infections, resistance to both drugs has been reported. In this study, the molecular characterization of spontaneous cefiderocol-resistant variants, a CR *A. baumannii* strain with antagonism to sulbactam, and an *A. lwoffii* strain with antagonism to avibactam, provides valuable insights into the mechanisms of resistance to cefiderocol. Some mechanisms observed are associated with mutations affecting efflux pumps, regulators, and iron homeostasis genes. These findings highlight the importance of understanding resistance mechanisms to optimize treatment options. They also emphasize the importance of early evaluation of drug synergies to address the challenges of antimicrobial resistance in *Acinetobacter* infections.

## INTRODUCTION

The widespread dissemination of Gram-negative bacteria (GNB) resistant to virtually all available antibiotics raises significant concerns (1, 2). Carbapenem-resistant (CR) *Acinetobacter* is especially problematic and the WHO and the Centers for Disease Control and Prevention (CDC) have recently categorized it as a critical priority pathogen (1, 3). The global emergence and widespread prevalence of *Acinetobacter* strains resistant to multiple antibiotic classes underscore the pressing demand for new antimicrobial therapies (2–4). Despite significant efforts by diverse research entities and pharmaceutical companies over the past decade (5–7), the approval of novel drugs targeting CR *Acinetobacter* has been limited. Only two drugs—cefiderocol and sulbactam-durlobactam—have received approval from the U.S. Food and Drug Administration (FDA) for treating *Acinetobacter baumannii-calcoaceticus* complex infections (https://www.accessdata.fda.gov/drugsatfda_docs/label/2019/209445s000lbl.pdf, https://www.accessdata.fda.gov/drugsatfda_docs/label/2023/216974Orig1s000Correctedlbl.pdf).

Cefiderocol, a siderophore cephalosporin, includes a catechol-type siderophore group (aminoacyl catechol) bound through a di-methylene linker to the C3 position of the dihydrothiazine ring. The catechol moiety chelates ferric iron (Fe^3+^) and facilitates uptake through GNB outer membranes using the TonB-dependent iron transport system (8). The cefiderocol unique properties also enhance its stability against β-lactamases (9, 10).

Cefiderocol, which is suggested as part of a combination therapy regimen when used against CR *A. baumannii* (CRAB) (11), should be reserved as a last line antibiotic for use in cases where other antibiotics have failed or no other treatments are available.

*In vitro* susceptibility to cefiderocol testing carried out in combination with sulbactam or avibactam showed a reduction of minimum inhibitory concentration (MIC) values in cefiderocol-non-susceptible isolates (12). *In vivo* models demonstrated efficacy for these combinations against all evaluated cefiderocol-non-susceptible CRAB isolates (13). Avibactam, a non-β-lactam β-lactamase inhibitor, reduced resistance to cefiderocol by suppressing cefiderocol-hydrolyzing secondary enzymes like Vietnamese extended-spectrum β-lactamase (VEB) and *Pseudomonas* extended resistance (PER), potentially present in CRAB isolates (14). Sulbactam, known for weakly inhibiting intrinsic *Acinetobacter*-derived cephalosporinases (ADCs), could provide activity in this combination via ADC inhibition with its high exposure (13).

Synergy, the effect observed when the combination of two antibiotics is greater than the sum of their individual effects, has been shown to be a successful alternative to preserve available drugs (15–18). However, antagonism, the effect seen when the combination of two antibiotics is less effective than one of the antibiotics alone, potentially diminishing treatment efficacy, has also been observed (16). Understanding these interactions is crucial for optimizing antibiotic therapy and combating resistant bacterial infections. In this study, we characterize at the molecular level the unexplained antagonism between cefiderocol and β-lactamase inhibitors of two CR *Acinetobacter*: a CRAB strain displaying antagonism with sulbactam and an *A. lwoffii* strain showing antagonism with the combination of cefiderocol plus avibactam. The insights gained from this research enhance our understanding of the antagonistic phenomenon and serve as a vital warning about the importance of pre-evaluating these combinations to prevent potential adverse impacts on clinical outcomes.

## MATERIALS AND METHODS

### Bacterial strains

**Fig 1:**
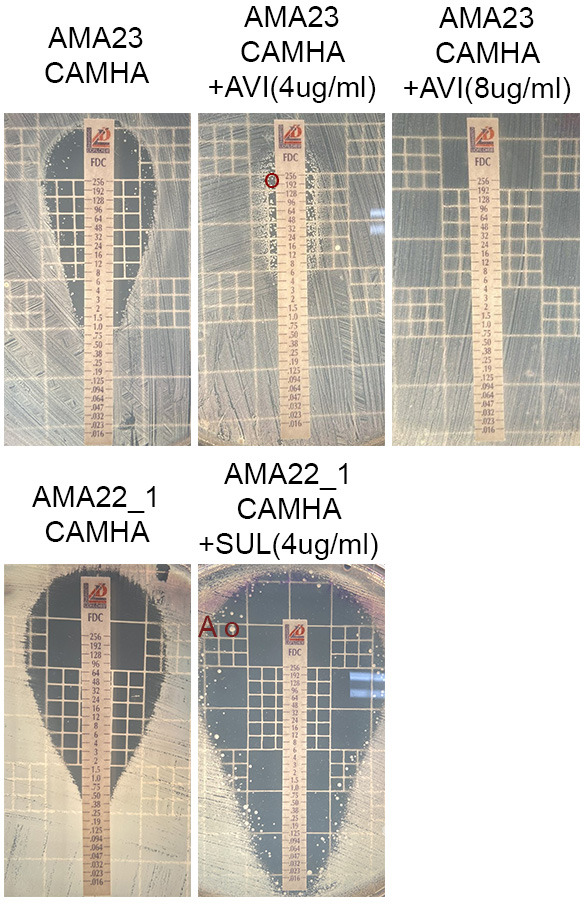
Effect of β-lactamase inhibitors [avibactam (AVI) or sulbactam (SUL)] on the antimicrobial susceptibility of *Acinetobacter* spp. to performed cefiderocol susceptibility. Minimum inhibitory concentration (MIC) was performed following manufacturer's recommendations (Liofilchem S.r.l., Italy). Red circles indicate intracolonies chosen for this study (AMA22_1R, AMA23_4R, and AMA23_8R). CAMHA: cation-adjusted Mueller-Hinton agar.

The carbapenem-resistant clinical *Acinetobacter* isolates AMA22_1 (*A. baumannii*) and AMA23 (*A. lwoffii*), both containing *bla*_NDM-1_, were used in this study (12, 19). In addition, spontaneous cefiderocol-resistant variants (AMA22_1R and AMA23_4R, and AMA23_8R of the AMA22_1 and AMA23 parental strains), which have emerged within the inhibition ellipse zones of cefiderocol in cation-adjusted Mueller-Hinton agar (CAMHA) containing 4 µg/mL of sulbactam or 4–8 µg/mL avibactam, respectively, were also used (Fig. 1).

The selected mutant variants of both strains showed different levels of cefiderocol susceptibility. All selected mutants were stored at −80°C as Luria Bertani (LB) broth containing 20% glycerol stocks. The stability of the different levels of cefiderocol resistance in the mutant variants was performed by daily subcultures in antibiotic free plates.

### Antimicrobial susceptibility testing (AST)

The antibiotic susceptibility assays were carried out in accordance with the Clinical and Laboratory Standards Institute (CLSI) guidelines (20). AMA22_1, AMA23, and its corresponding variants cells were cultivated in CAMHA or iron-depleted CAMHB, when corresponded and adjusted to a 0.5 McFarland standard value, and were utilized for the assays. The iron content was confirmed to be ≤0.03 mg/L in the iron-depleted CAMHB used in the AST as determined using the Iron Assay Kit (Sigma-Aldrich, MA, United States) following the manufacturer's recommendations. Disks and commercial E-strips (Liofilchem S.r.l., Roseto degli Abruzzi, Italy) containing varying concentrations of different antibiotics were employed. These included imipenem (IMI), meropenem (MRP), ceftazidime (CAZ), ceftriaxone (CRO), gentamicin (GM), ciprofloxacin (CIP), ceftazidime-avibactam (CZA), cefiderocol (FDC), ampicillin (AMP), ampicillin-sulbactam (AMS), and amikacin (AN). Cefiderocol MICs were also performed by two different dilution methods, comASP (Liofilchem S.r.l., Roseto degli Abruzzi, Italy) and in-house broth microdilution (BMD) with ID-CAMHB. All commercial assays strictly followed the manufacturer's instructions (https://www.liofilchem.com/images/brochure/mic_test_strip_patent/MTS51.pdf).

Additionally, in specific cases, CAMHA medium was supplemented with 1, 2, 4, 8, and 16 µg/mL of sulbactam or avibactam (Sigma-Aldrich). Measurements were taken after incubating the plates at 37°C for 18 hours. Interpretation of the data was done based on CLSI breakpoints (20), with *Escherichia coli* ATCC 25922 employed for quality control purposes. Each susceptibility assay was repeated at least twice using independent biological samples on each occasion.

### Whole genome sequence analysis

The genomic DNA extraction of the parental strains (AMA22_1 and AMA23) and the three randomly selected variants (AMA22_1R and AMA23_4R, and AMA23_8R) was performed following the manufacturer's instructions using the Wizard Promega kit (Promega, Madison, WI, USA). For whole genome sequencing, SEQCENTER sequencing service (Pittsburgh, PA, USA) performed the outsourced procedure utilizing NextSeq 550 Illumina technology. To ensure sequence quality, we conducted FASTQC software analysis (https://www.bioinformatics.babraham.ac.uk/projects/fastqc/), followed by trimming and filtering using Trimmomatic software (version: 0.40, ILLUM-NACLIP: TrueSeq3-PE.fa.2:30:10; LEADING:3; TRAILING:3; SLIDINGWINDOW: 4:15; MINLEN:36) (21). *De novo* sequence assembly was carried out using SPAdes (version: 3.15.4, default parameters) (22) and subsequently assessed for quality using QUAST (version: 5.2.0) (23). Genome annotation was accomplished via PROKKA (24), whereas variant calling employed the *breseq* and *gdtools* software packages (version: 0.38.1, consensus mode, default parameters) (25). Recombination regions were detected and eliminated using Gubbins software (version: 3.3.0, default parameters) (26). The raw genomic sequencing data and assemblies have been deposited in the Zenodo repository (https://zenodo.org/records/10729558).

### RNA extraction and transcriptional analysis through RT-qPCR

For RNA extractions, overnight cultures of AMA22_1, AMA23, AMA22_1R, AMA23_4R, and AMA23_8R strains underwent a 1:10 dilution in iron-depleted CAMHB and were incubated with agitation at 200 rpm for 18 hours at 37°C. RNA extraction from each sample utilized the Direct-zol RNA Kit (Zymo Research, Irvine, CA, USA) following the manufacturer's protocol. RNA extractions were done in triplicates. Subsequently, the DNase-treated RNA was used to synthesize cDNA employing the iScriptTM Reverse Transcription Supermix for qPCR reagents (Bio-Rad, Hercules, CA, USA) following the provided manufacturer's guidelines. The concentration of the resulting cDNA was adjusted to 50 ng/µL and 2 µL of the adjusted cDNA was used to carry out qPCR reaction using qPCRBIO SyGreen Blue Mix Lo-ROX according to the manufacturer's instructions (PCR Bio-systems, Wayne, PA, USA).

**Fig 2:**
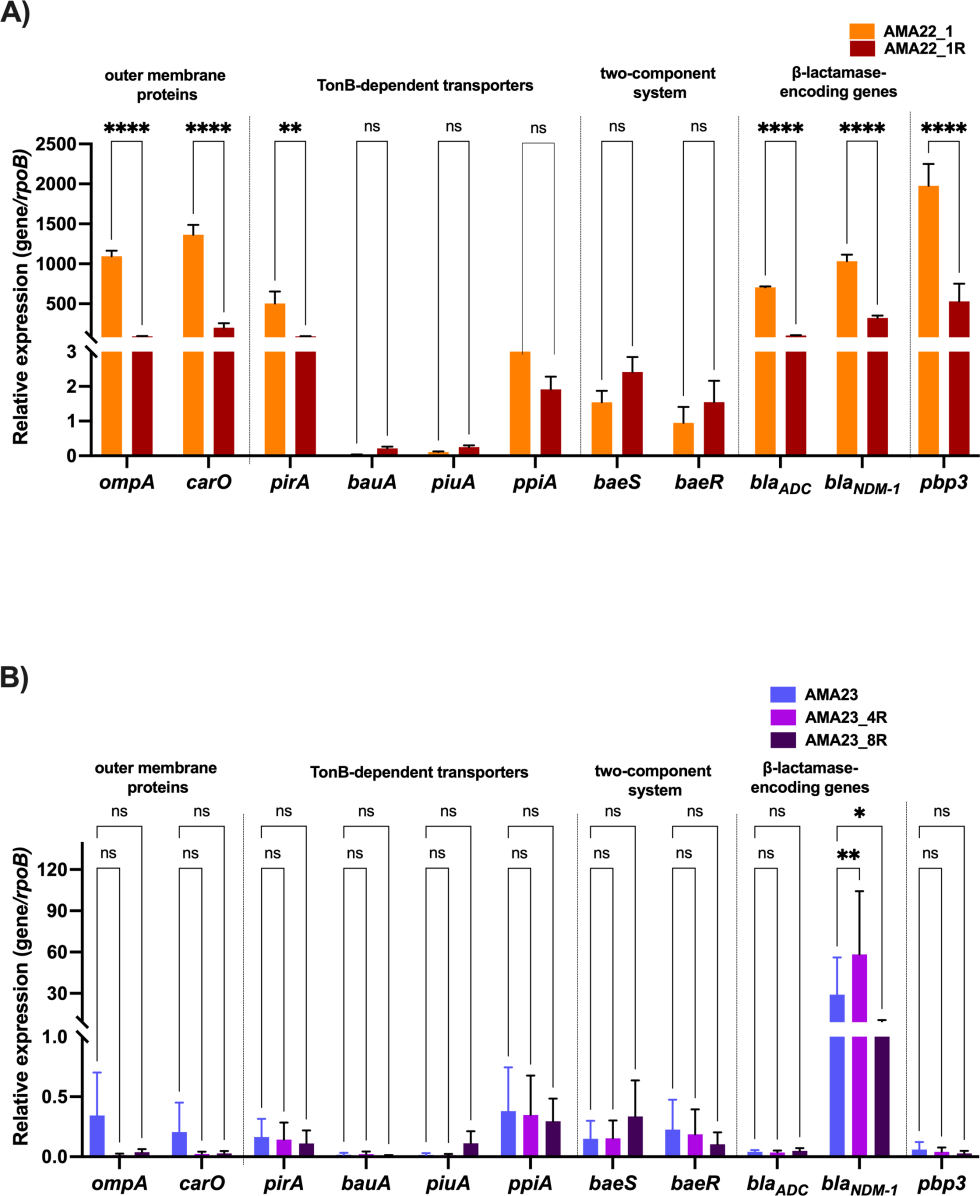
Expression of genes coding for outer membrane proteins (*ompA* and *carO*), TonB-dependent transporters and iron homeostasis (*pirA*, *bauA*, *piuA,* and *ppiA*), the BaeRS two-component system, β-lactamase and *pbp3* in the AMA22_1 and AMA22_1R (**A**) or AMA23, AMA23_4R, and AMA23_8R (**B**) strains. The data shown of qRT-PCR are mean ± SD. Fold changes were calculated using ΔCt analysis. At least three independent biological samples were tested using four technical replicates. Statistical significance (*P* < 0.05) was determined by two-way ANOVA followed by Tukey's multiple comparison test. Significance was indicated by: *P < 0.05, **P < 0.01, ***P < 0.001, and **** *P* < 0.0001.

In Table S1, the primers used for the transcriptional analysis are listed. Each cDNA was tested independently in triplicate utilizing the CFX96 TouchTM Real-Time PCR Detection System (Bio-Rad, Hercules, CA, USA). The transcript levels of each sample were standardized against the *rpoB* transcript levels in individual cDNA samples (27). The quantification of gene expression was conducted using the comparative threshold method 2^-ΔCt^ (28) (Fig. 2) and the 2^-ΔΔCt^ method (Table S6). Statistical differences were determined using analysis of variance (ANOVA) followed by Tukey's multiple comparison test (*P* < 0.05) employing GraphPad Prism (GraphPad software, San Diego, CA, USA).

## RESULTS AND DISCUSSION

### Spontaneous occurrence of cefiderocol resistance in the presence of β-lactamase inhibitors

In an experiment testing the antibiotic cefiderocol with β-lactamase inhibitors such as avibactam, relebactam, zidebactam, and sulbactam, intracolonies were observed within the inhibition zones for two carbapenem-resistant *Acinetobacter* strains (AMA23 and AMA22_1). This growth occurred when either sulbactam or avibactam was used at a concentration of 4 µg/mL in cation-adjusted Mueller-Hinton agar (CAMHA) (13). Two randomly selected colonies from plates containing strains AMA22_1 and AMA23 (AMA22_1R and AMA23_4R) were selected for further studies (Fig. 1A). Additionally, for the AMA23 strain, high cefiderocol resistance (>256 µg/mL) was noted when testing was carried out in the presence of increasing concentrations of avibactam. Cells from the plate containing 8 µg/mL were selected for WGS (AMA23_8R) (Fig. S1). The MIC of cefiderocol, measured using three different methods—E-strips, comASP, and broth microdilution assays—was higher for all three mutants compared to their parental strains (Table 1).

**TABLE 1:** Cefiderocol minimum inhibitory concentration (MIC) of *A. baumannii* AMA22_1, *A. lwoffii* AMA23, and the isogenic mutant variants obtained within the inhibition ellipse zones of cefiderocol containing 4 µg/mL of sulbactam or 4–8 µg/mL avibactam, respectively, using gradient strips and microdilution assays^*a*^

Strain	BMD
AMA22_1	2
AMA22_1R	4
AMA23	1
AMA23_4R	16
AMA23_8R	8

^*a*^ BMD: Broth microdilution using ID-CAMHB. All experiments were performed in triplicates.

We tested increasing concentrations of sulbactam and avibactam on AMA22_1 and AMA23, respectively, to determine whether the emergence of spontaneous cefiderocol-resistant colonies depended on the concentration of β-lactamase inhibitors. As previously observed, colonies grew within the zone of inhibition on plates containing 4 µg/mL of sulbactam or avibactam (Fig. S1). In the case of strain AMA23, the MIC values in plates supplemented with 8 µg/mL were >256 µg/mL (Fig. S1; Table 2). *In vitro* assessment revealed a paradoxical effect of avibactam concentration on cefiderocol in AMA23, demonstrating synergy at concentrations below 2 mg/L or above 16 mg/L. However, it exhibited antagonism at concentrations near the breakpoint.

**TABLE 2:** Cefiderocol minimum inhibitory concentration (MIC) values of AMA22_1 and AMA23 in cation-adjusted Mueller Hinton agar (CAMHA) containing increases in concentration of sulbactam or avibactam, respectively, using gradient strips^*a*^

Cefiderocol MICs (E-strips)			
Drug and cc (ug/mL)	AMA22_1	Drug and cc (ug/mL)	AMA23
CAMHA 0 µg/mL SUL	0.5	CAMHA 0 µg/mL AVI	1^*b*^
CAMHA + 1 µg/mL SUL	0.19	CAMHA + 1 µg/mL AVI	0.5
CAMHA + 2 µg/mL SUL	0.047	CAMHA + 2 µg/mL AVI	0.75
CAMHA + 4 µg/mL SUL	48^*b*^	CAMHA + 4 µg/mL AVI	>256
CAMHA + 8 µg/mL SUL	0.023	CAMHA + 8 µg/mL AVI	>256
CAMHA + 16 µg/mL SUL	<0.016	CAMHA + 16 µg/mL AVI	0.5

^*a*^ SUL= sulbactam, AVI= avibactam.

^*b*^ Occurrence of intracolonies within the inhibition ellipse zones of cefiderocol. All experiments were performed in triplicates.

Evaluation of increasing sulbactam concentrations in AMA22_1 was limited to 8 mg/L or less due to observed growth difficulties beyond this threshold (16 µg/mL)—suggesting values nearing or surpassing the MIC. Nonetheless, at lower concentrations, a similar paradoxical effect was noted, with antagonism observed near the breakpoint.

All three mutant strains were subcultured 10 consecutive days and no reduction of cefiderocol resistance levels was observed, demonstrating the inheritable nature of the acquired trait.

The susceptibility of AMA22_1R, AMA23_4R, and AMA23_8R to various antibiotics (meropenem, imipenem, gentamicin, ampicillin/sulbactam, amikacin, ciprofloxacin, levofloxacin, tigecycline, colistin, and trimethoprim-sulfamethoxazole) was evaluated to identify any collateral sensitivity or cross-resistance to cefiderocol (Table S2). No differences in resistance profiles were observed between the parental strain and the corresponding variants for most of the antibiotics tested. Collateral susceptibility to ampicillin and amikacin was observed in AMA23 variants (Table S2), which was confirmed by MIC determination showing a twofold and fourfold decrease dilution, respectively (Table S3). A slight cross-resistance for meropenem and imipenem was observed in AMA23_8R compared to the parental strain. No major differences in resistant profiles were observed between the parental strain and the corresponding variants.

While the clinical significance of this phenomenon remains unclear, recent research has illuminated key aspects of cefiderocol heteroresistance. Notably, observations of small colonies within inhibition zones reverting to their original form upon drug removal suggest the instability of cefiderocol heteroresistance (29). Additionally, strains harboring multiple classes of β-lactamase resistance genes may see restored activity with the addition of β-lactamase inhibitors like ceftazidime/avibactam to cefiderocol (29). Moreover, animal models have shown that combining ceftazidime-avibactam can impede the development of *in vivo* resistance to cefiderocol, while *in vitro*, microcolonies within the inhibition zone notably decreased for *A. baumannii* isolates with high-end susceptibility (MIC = 2 mg/L) (13). Most of these studies have primarily examined *A. baumannii* for the occurrence of cefiderocol heteroresistance, with most showing reversion of the observed resistance in the absence of cefiderocol (29–31). Here, we observed that heteroresistant colonies maintained their properties after serial passage in antibiotic-free media, and inhibitors at typical assay concentrations did not reverse the resistance. This phenomenon was not only observed in *A. baumannii*, but also in other CR *Acinetobacter* species.

### Distinct changes at the genome level of the selected spontaneous resistant variants

All three resistant variants, AMA22_1R, AMA23_4R, and AMA23_8R, were subjected to genomic analysis. The AMA22_1R nucleotide substitutions revealed eight synonymous and three non-synonymous mutations. Of the non-synonymous mutations, two affected genes encoding hypothetical protein (AMA22N_02150), while one impacted the gene encoding the transposase IS*Aba26* (AMA22N_03871) (Table S4). Additionally, a deletion of two nucleotides was identified in the gene encoding a hypothetical protein, and an insertion of two nucleotides was found in the gene encoding the cobalt-zinc-cadmium resistance protein CzcA. These genes are components of an efflux pump belonging to the RND group, a superfamily that includes various efflux pumps associated with multidrug resistance phenotypes, such as the AdeABC efflux pump. While CzcA is a part of the CzcCBA efflux pump (32), which confers resistance to heavy metals, its role in multidrug resistance remains unexplored. Investigating the association of CzcCBA with multidrug resistance will be crucial for understanding its function in this context. None of intergenic changes were found in regulator or promoter regions (Table S5).

The analysis of the nucleotide substitutions of AMA23_4R and AMA23_8R revealed 18 and 15 synonymous mutations, respectively. Three non-synonymous mutations, identical to both isolates, affected genes encoding the BfmR DNA-binding transcriptional regulator and two transposases (IS*66* (AMA23_02604) and IS*Aba31* (AMA23_02787) (Table S4). BfmR is involved in regulating biofilm formation and has also been associated with enhanced meropenem resistance (33–35). These mutations not only have the potential to influence meropenem resistance (36) but also may impact a wide range of β-lactam antibiotics, including cefiderocol.

An insertion of 17 nucleotides was identified in a gene coding for a transposase (IS*Aba125*). Mutations within insertion sequences can affect the functionality of downstream genes' promoters/enhancers and the transposition mechanism (37). None of the insertion sequences harboring mutations were observed to affect genes related to iron uptake/homeostasis or resistance to β-lactams and cefiderocol. Additionally, 19 and 23 intergenic changes were found in AMA23_4R and AMA23_8R, respectively. None of intergenic changes were found in regulator or promoter regions (Table S5).

### Modifications in the transcript levels of key genes in the spontaneous resistant variants

We extracted and quantified mRNA from AMA22_1, AMA23, and IHC cells to assess the expression levels of genes related to outer membrane proteins, TonB-dependent transporters and iron homeostasis, the two-component system BaeRS, β-lactamase, and *pbp3*. Figure 2A shows significant down-regulation of *ompA*, *carO*, β-lactamase genes, and *pbp3* in the AMA22_1R compared to the parental strain. Conversely, genes encoding BaeRS did not exhibit differences. Interestingly, of the iron-uptake related genes studied, *pirA* was found to be down-regulated (Fig. 2A; Table S6). In AMA23 cells, the genes encoding outer membrane proteins (*ompA* and *carO*) showed down-regulation in both mutant variant strains (Fig. 2B; Table S6). Notably, an increased expression of *bla*_NDM-1_ was observed in AMA23_4R (Fig. 2B; Table S6). There were no significant differences noted in the TonB-dependent transporters and iron homeostasis for AMA23 cells and gene encoding for the two-component system BaeSR (Fig. 2B; Table S6).

Despite the presence of a mutation in the gene encoding the transcriptional regulator BfmR AMA23 variant cells, evaluation of transcriptional expression levels of BfmRS encoding genes (Fig. S2; Table S5) showed no differences (Fig. S2; Table S6).

Previous reports have indicated significant alterations in the expression levels of genes related to iron-uptake systems, β-lactam resistance, and biofilm formation in heteroresistant variants of a CRAB strain, particularly in cultures supplemented with cefiderocol and human fluids (34). Additionally, mutations in the *baeSR* genes were associated with reduced susceptibility of *A. baumannii* to cefiderocol by up-regulating the expression of the MFS family efflux pump and MacAB-TolC efflux pump (38). The modified expression of these genes seen in the studied heteroresistant cells, AMA22_1R, AMA23_4R, and AMA23_8R, could potentially be linked to the increased cefiderocol MIC seen in these strains.

The complexity of the regulation of gene expression known to contribute to cefiderocol resistance in different *Acinetobacter* species and mutant variants can explain the different levels observed for some of the genes evaluated. However, several genes implicated in bacterial permeability, such as *ompA* and *carO*, along with genes associated with TonB iron uptake systems, such as *pirA*, and two-component system consistently exhibited a similar pattern across all studied variants, contributing to resistance to cefiderocol.

The identified limitations of this work include the limited number of strains analyzed and the fact that RNA extraction was performed using overnight cultures that were not exposed to cefiderocol. Future experiments should include a larger number of strains as well as bacteria cultured to mid-log phase treated with or without 0.5× or 1× MIC of cefiderocol to better delineate the molecular drivers of resistance.

### Concluding remarks

This study highlights the complex nature of emergent resistance to cefiderocol in *Acinetobacter* spp. Unlike some antibiotics, where resistance is acquired through the horizontal transfer of one or more genes, the resistance observed here is likely due to multiple genomic mutations that alter the expression of various genes, leading to phenotypic changes. These findings suggest that resistance is driven by multiple mechanisms. Additionally, the observed antagonistic effect when cefiderocol is combined with avibactam or sulbactam indicates intricate interactions resulting from these genotypic and phenotypic changes. Such interactions create a complex network that can influence the efficacy of combination therapies involving cefiderocol, making it challenging to predict therapeutic outcomes. Therefore, it is crucial to evaluate potential synergies or antagonisms on a case-by-case basis to avoid undesirable clinical results.

## Data Availability

The genomic sequencing data and assemblies have been deposited in the Zenodo repository (https://zenodo.org/records/10729558).

## SUPPLEMENTAL MATERIAL

Supplemental figures and tables spectrum.00930-24-s0001.pdf Fig. S1 and S2; Tables S1 to S6.

ASM does not own the copyrights to Supplemental Material that may be linked to, or accessed through, an article. The authors have granted ASM a non-exclusive, world-wide license to publish the Supplemental Material files. Please contact the corresponding author directly for reuse.
