# Supplementary material for: Hetero-antagonism of avibactam and sulbactam with cefiderocol in carbapenem-resistant Acinetobacter spp
Source: Microbiol Spectr. 2024 Aug 20;12(10):e00930-24. doi: 10.1128/spectrum.00930-24 (PMC11448186; doi:10.1128/spectrum.00930-24)
Supplement: Supplemental figures and tables — Fig. S1 and S2; Tables S1 to S6. [file spectrum.00930-24-s0001.pdf]

Supplementary Material Legends

Figure S1. Effect of β-lactamase inhibitors sulbactam (SUL) for AMA22_1 (A) and avibactam (AVI) for AMA23 (B) on the antimicrobial susceptibility to cefiderocol. Minimum inhibitory concentration (MIC) on cation adjusted Mueller-Hinton agar supplemented with 1 to 16 µg/mL SUL or AVI was performed following manufacturer's recommendations (Liofilchem S.r.l., Italy).

Figure S2. Expression of genes coding for the two-component regulatory system BfmRS in the AMA23, AMA23_4R, and AMA23_8R strains. The data shown of qRT-PCR are mean ± SD. Fold changes were calculated using double ΔCt analysis. At least three independent biological samples were tested using four technical replicates. Statistical significance ($p < 0.05$) was determined by two-way ANOVA followed by Tukey's multiple comparison test. Significance was indicated by: * $p < 0.05$, ** $p < 0.01$, *** $p < 0.001$ and **** $p < 0.0001$.

Table S1. Primers used for the transcriptional analysis of this work.

Table S2. Antimicrobial susceptibility of the wild-type and the hetero-resistant selected strains.

Table S3. Antimicrobial susceptibility of the CRAB AMA23 and the heteroresistant variants, performed using e-strips on Mueller Hinton Agar.

Table S4. Mutational analysis of AMA22_1R compared to the parental strain.

Table S5. Mutational analysis of AMA23_4R, and AMA23_8R compared to the parental strain.

Table S6. Comparison of the level of expression of genes obtained by qRT-PCR in the AMA22 and AMA23 variants compared to the corresponding wild-type strains using double ΔCt analysis.

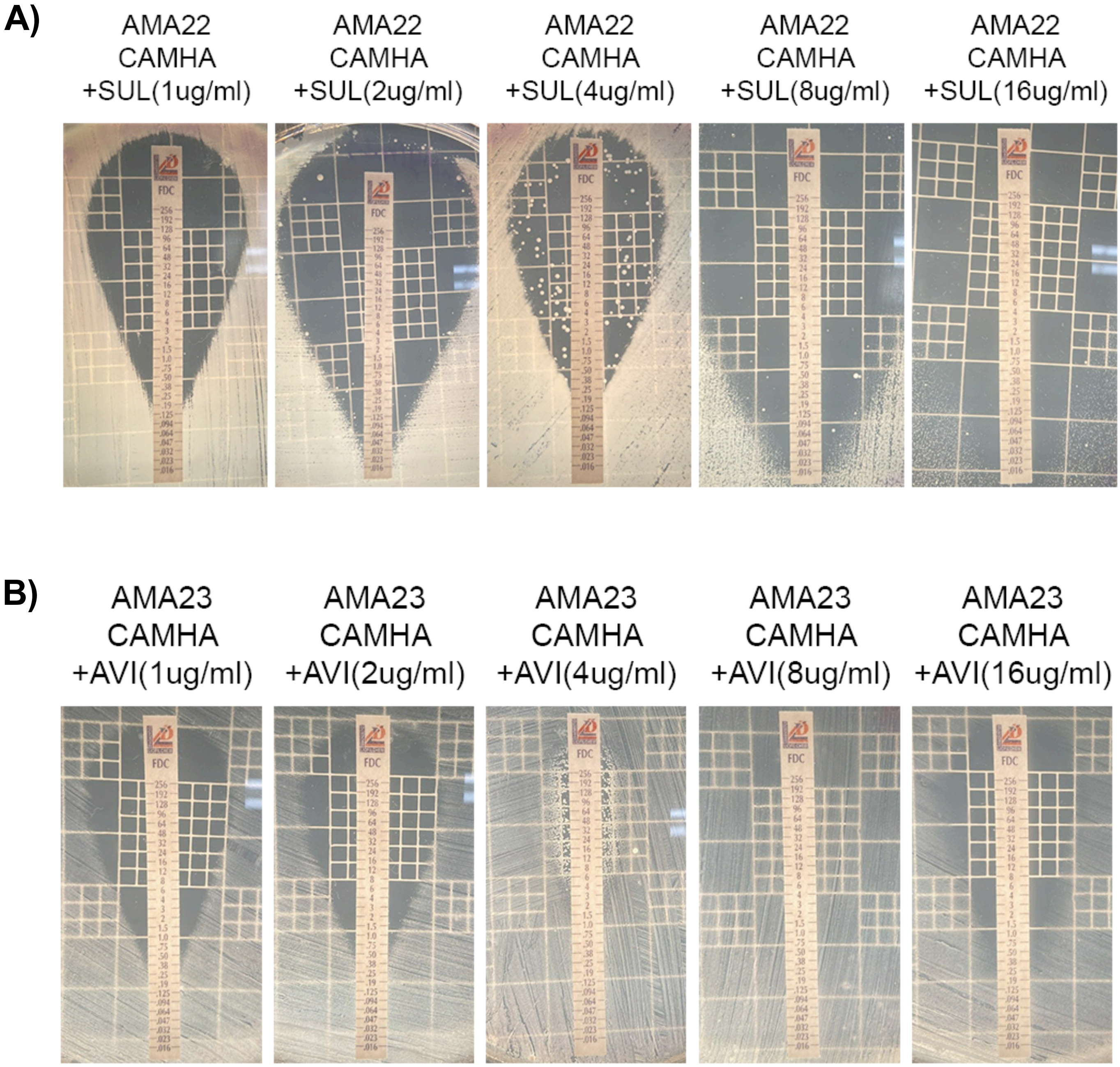
A)
AMA22 CAMHA +SUL(1ug/ml)
AMA22 CAMHA +SUL(2ug/ml)
AMA22 CAMHA +SUL(4ug/ml)
AMA22 CAMHA +SUL(8ug/ml)
AMA22 CAMHA +SUL(16ug/ml)
B)
AMA23 CAMHA +AVI(1ug/ml)
AMA23 CAMHA +AVI(2ug/ml)
AMA23 CAMHA +AVI(4ug/ml)
AMA23 CAMHA +AVI(8ug/ml)
AMA23 CAMHA +AVI(16ug/ml)

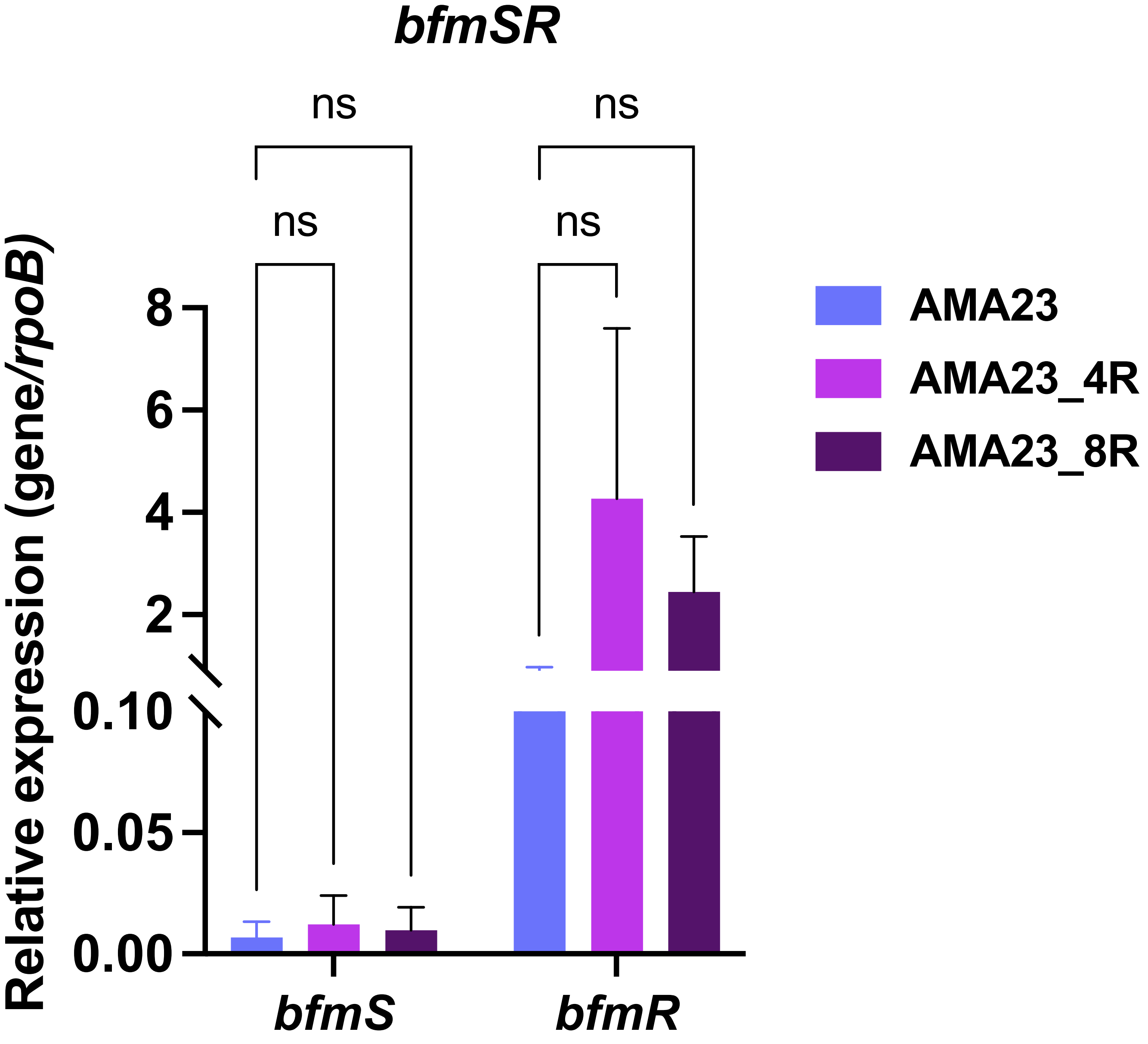
bfmSR
ns
ns
ns
ns
Relative expression (gene/rpoB)
8
6
4
2
0.10
0.05
0.00
bfmS
bfmR
AMA23
AMA23_4R
AMA23_8R

Table S1: List of primers used in the study to perform RT-qPCR

Primer name	5'-sequence-3'
ompA-F	TCTTGGTGGTCACTTGAAGC
ompA-R	ACTCTTGTGGTTGTGGAGCA
carO-F	GGCGGATGAAGCTGTTGTTC
carO-R	GCCATAACAAAGCACCACCG
pirA-F	GTCTATGGCTTTTGCTGCACA
pirA-R	GCGATTGCTTCACTTGCTCT
bauA-F	AAATGTTTGGCCGCGTTGAGGT
bauA-R	CAATCGTGCAAACGGTTCATCAGC
piuA-F	TTGGTGTACCTCGCTCAATG
piuA-R	AGTCTGGGCGTGCTTGTAAA
ppiA-F	CGATGATAAAGCACCAATGAGTGT
ppiA-R	TCGCCAAAGTACCTCGTGTA
baeS-F	GGTCTTGCTTACCACGCTGT
baeS-R	AATCATCTGGTGCTGCCGTT
baeR-F	AGTCGAGTTAGCGCATCTGG
baeR-F	TCTTCAGTACGTGCCGTCAC
blaADC-F	TGCCAACCTTAACCCACA
blaADC-R	AGTTTGTAACGTTGCCGG
blaNDM-1-F	GAAGCTGAGCACCGCATTAG
blaNDM-1-R	AACCAGATCGCCAAACCGTT
pbp3-F	AGCGAACAAAGCAAACACGA
pbp3-R	GCCCAGAGCAGATAAAACCG

Table S2: Antimicrobial susceptibility of the CRAB AMA22, AMA23 and the heteroresistant variants, performed using disk diffusion (Liofilchem S.r.l., Italy) on Mueller Hinton Agar.

Disk	AMA22_1	AMA22_1R	AMA23	AMA23_4R	AMA23_8R
Cefiderocol (FDC) 30ug	20	20	34	32	33
Imipenem (IMI) 10ug	12	13	21	17	18
Meropenem (MRP) 10ug	7	7	16	15	13
Ceftazidime (CAZ) 30ug	6	6	6	6	6
Amikacin (AN) 30ug	22	24	25	30	28
Ceftriaxone (CRO) 30ug	6	6	13	15	15
Gentamicin (GM) 10ug	28	29	40	41	41
Ciprofloxacin (CIP) 5ug	33	31	43	44	44
Ampicillin (AMP) 10ug	6	6	13	18	17
Ceftazidime-AVI (CZA) 14ug	7	7	33	32	32
Ampicillin-SUL (AMS) 20ug	9	10	32	33	32

Table S3. Antimicrobial susceptibility of the CRAB AMA23 and the heteroresistant variants, performed using e-strips on Mueller Hinton Agar.

Strain	Amikacin (mg/L)	Ampicillin (mg/L)
AMA23	24	>256
AMA23_4R	1.5	96
AMA23_8R	1.5	96

Table S4. Mutational analysis of AMA22_1R compared to the parental strain.

Predicted mutations of AMA22_1 intrahalo colonies				
**position**	**mutation**	**annotation**	**gene**	**description**
658418	+50 bp	noncoding (73/74 nt)	*AMA22N_00629* ←	tRNA-Asp
2039680	Δ2 bp	coding (665-666/1110 nt)	*AMA22N_01993* →	hypothetical protein
2205235	A→C	I308L (ATT→CTT)	*AMA22N_02150* →	hypothetical protein
2205280	G→C	A323P (GCT→CCT)	*AMA22N_02150* →	hypothetical protein
3208944	Δ1 bp	noncoding (1/77 nt)	*AMA22N_03103* →	tRNA-Pro
3469078	2 bp→A	coding (1604-1605/3159 nt)	*czcA_2* →	Cobalt-zinc-cadmium resistance protein CzcA
3940075	A→T	intergenic (+65/-29)	*acuI* → / → *AMA22N_03871*	Acrylyl-CoA reductase AcuI/IS256 family transposase ISAba26
3940077	T→A	intergenic (+67/-27)	*acuI* → / → *AMA22N_03871*	Acrylyl-CoA reductase AcuI/IS256 family transposase ISAba26
3940087	A→T	intergenic (+77/-17)	*acuI* → / → *AMA22N_03871*	Acrylyl-CoA reductase AcuI/IS256 family transposase ISAba26
3940089	A→C	intergenic (+79/-15)	*acuI* → / → *AMA22N_03871*	Acrylyl-CoA reductase AcuI/IS256 family transposase ISAba26
3940092	G→A	intergenic (+82/-12)	*acuI* → / → *AMA22N_03871*	Acrylyl-CoA reductase AcuI/IS256 family transposase ISAba26
3940107	T→G	Y2D (TAT→GAT)	*AMA22N_03871* →	IS256 family transposase ISAba26
3940115	T→A	A4A (GCT→GCA)	*AMA22N_03871* →	IS256 family transposase ISAba26
3940133	A→T	A10A (GCA→GCT)	*AMA22N_03871* →	IS256 family transposase ISAba26
3940136	T→C	A11A (GCT→GCC)	*AMA22N_03871* →	IS256 family transposase ISAba26
3940142	A→G	L13L (TTA→TTG)	*AMA22N_03871* →	IS256 family transposase ISAba26
3940152	T→C	L17L (TTA→CTA)	*AMA22N_03871* →	IS256 family transposase ISAba26
3940160	T→A	T19T (ACT→ACA)	*AMA22N_03871* →	IS256 family transposase ISAba26
3940163	T→A	P20P (CCT→CCA)	*AMA22N_03871* →	IS256 family transposase ISAba26
4042959	A→T	V8V (GTA→GTT)	*AMA22N_04003* →	IS3 family transposase ISAba14

Variant calling was performed using breseq and gdtools software (Parameter: Default)
Reference sequence: A. baumannii AMA22_1 without beta-lactam inhibitor

Table S5. Mutational analysis of AMA23_4R, and AMA23_8R compared to the parental strain.

Predicted mutations of AMA23 intrahalo colonies						
position	mutation	AMA23_4R	AMA23_8R	annotation	gene	description
161,533	G→C	1	1	intergenic (-96/-232)	*AMA23_00159* ← / → *AMA23_00160*	hypothetical protein/hypothetical protein
284,575	C→A	1	1	V105V (GTC→GTA)	*AMA23_00272* →	IS1 family transposase ISPa14
284,581	2 bp→GT	0	1	coding (321-322/702 nt)	*AMA23_00272* →	IS1 family transposase ISPa14
302,195	A→C	0	1	intergenic (+284/-537)	*AMA23_00290* → / → *mlaA*	hypothetical protein/putative phospholipid-binding lipoprotein MlaA
302,197	2 bp→AT	0	1	intergenic (+286/-534)	*AMA23_00290* → / → *mlaA*	hypothetical protein/putative phospholipid-binding lipoprotein MlaA
317,447	A→G	1	0	R215R (AGA→AGG)	*AMA23_00302* →	IS4 family transposase ISCja2
317,456	G→T	1	0	S218S (TCG→TCT)	*AMA23_00302* →	IS4 family transposase ISCja2
317,483	C→A	1	1	P227P (CCC→CCA)	*AMA23_00302* →	IS4 family transposase ISCja2
317,501	C→T	1	0	G233G (GGC→GGT)	*AMA23_00302* →	IS4 family transposase ISCja2
551,275	98 bp→47 bp	0	1	intergenic (-3/+1)	*AMA23_00526* ← / ← *AMA23_00527*	tRNA-Asp/tRNA-Asp
1,067,181	Δ100 bp	1	1	intergenic (-211/+207)	*AMA23_01038* ← / ← *panM*	tRNA-Phe/PanD maturation factor
1,206,815	Δ100 bp	1	1	intergenic (+1/+387)	*AMA23_01174* → / ← *AMA23_01175*	tRNA-Pro/hypothetical protein
1,958,397	T→C	0	1	intergenic (-567/+174)	*AMA23_01883* ← / ← *aphA*	hypothetical protein/Aminoglycoside 3'-phosphotransferase
1,973,646	T→G	0	1	A114A (GCA→GCC)	*AMA23_01901* ←	IS66 family transposase ISAba25
2,089,991	98 bp→20 bp	1	1	intergenic (+2/-6)	*AMA23_02006* → / → *AMA23_02007*	tRNA-Gln/tRNA-Gln
2,275,902	Δ100 bp	1	1	intergenic (+2115/-721)	*suhB* → / → *mlaF*	Inositol-1-monophosphatase/putative phospholipid import ATP-binding protein MlaF
2,410,736	G→A	1	1	G102D (GGT→GAT)	*bfmR* →	bfmR/bfmS system is a bacterial two-component regulatory system consisting of the membrane sensor,
2,567,101	99 bp→21 bp	1	1	intergenic (-1/+1)	*AMA23_02494* ← / ← *AMA23_02495*	tRNA-Lys/tRNA-Lys
2,683,305	C→T	1	1	S80S (TCC→TCT)	*AMA23_02603* →	hypothetical protein
2,683,502	A→T	1	1	G19G (GGA→GGT)	*AMA23_02604* →	hypothetical protein
2,683,756	A→G	1	1	Q104R (CAA→CGA)	*AMA23_02604* →	hypothetical protein (IS66 transposase – TnpB)
2,683,766	A→G	1	1	V107V (GTA→GTG)	*AMA23_02604* →	hypothetical protein
2,683,788	T→C	1	1	intergenic (+7/-68)	*AMA23_02604* → / → *AMA23_02605*	hypothetical protein/hypothetical protein
2,683,793	G→C	1	1	intergenic (+12/-63)	*AMA23_02604* → / → *AMA23_02605*	hypothetical protein/hypothetical protein
2,702,962	G→A	1	0	L88L (CTG→CTA)	*AMA23_02620* →	hypothetical protein
2,738,196	T→C	1	1	intergenic (-160/-608)	*AMA23_02655* ← / → *rpoN*	IS481 family transposase ISNwi5/RNA polymerase sigma-54 factor
2,738,199	A→T	1	0	intergenic (-163/-605)	*AMA23_02655* ← / → *rpoN*	IS481 family transposase ISNwi5/RNA polymerase sigma-54 factor
2,874,265	T→C	1	1	S22S (TCT→TCC)	*AMA23_02787* →	IS5 family transposase ISAba31
2,874,271	T→C	1	1	H24H (CAT→CAC)	*AMA23_02787* →	IS5 family transposase ISAba31

2,874,283	C→T	1	1	N28N (AAC→AAT)	*AMA23_02787 →*	IS5 family transposase ISAba31
2,874,289	T→C	1	1	R30R (CGT→CGC)	*AMA23_02787 →*	IS5 family transposase ISAba31
2,874,661	C→T	1	1	D154D (GAC→GAT)	*AMA23_02787 →*	IS5 family transposase ISAba31
2,874,682	C→A	1	1	V161V (GTC→GTA)	*AMA23_02787 →*	IS5 family transposase ISAba31
2,874,730	A→G	1	1	R177R (AGA→AGG)	*AMA23_02787 →*	IS5 family transposase ISAba31
2,874,742	A→T	1	1	T181T (ACA→ACT)	*AMA23_02787 →*	IS5 family transposase ISAba31
2,874,765	T→C	1	1	I189T (ATA→ACA)	*AMA23_02787 →*	IS5 family transposase ISAba31
2,982,998	G→A	1	1	N16N (AAC→AAT)	*AMA23_02894 ←*	IS3 family transposase ISAba14
3,011,593	G→A	1	1	intergenic (-101/+216)	*putP_2 ← / ← mpl*	Sodium/proline symporter/UDP-N-acetylmuramate--L-alanyl-gamma-D-glutamyl-meso-2,6-diaminoheptandioate ligase
3,164,429	G→A	1	1	intergenic (-549/-273)	*AMA23_03055 ← / → higB-2*	IS3 family transposase ISAcsp5/Toxin HigB-2
3,203,760	Δ99 bp	1	1	intergenic (+1496/-1468)	*AMA23_03098 → / → AMA23_03099*	hypothetical protein/IS21 family transposase ISAba8
3,203,859	Δ99 bp	1	1	intergenic (+1595/-1369)	*AMA23_03098 → / → AMA23_03099*	hypothetical protein/IS21 family transposase ISAba8
3,239,065	90 bp→60 bp	0	1	intergenic (+1/-484)	*AMA23_03137 → / → AMA23_03138*	tRNA-Ala/IS3 family transposase ISAba18
3,239,318	T→A	1	1	intergenic (+254/-320)	*AMA23_03137 → / → AMA23_03138*	tRNA-Ala/IS3 family transposase ISAba18
3,239,327	+T	1	1	intergenic (+263/-311)	*AMA23_03137 → / → AMA23_03138*	tRNA-Ala/IS3 family transposase ISAba18
3,239,338	T→C	1	1	intergenic (+274/-300)	*AMA23_03137 → / → AMA23_03138*	tRNA-Ala/IS3 family transposase ISAba18
3,239,341	G→A	1	1	intergenic (+277/-297)	*AMA23_03137 → / → AMA23_03138*	tRNA-Ala/IS3 family transposase ISAba18
3,239,346	C→G	1	1	intergenic (+282/-292)	*AMA23_03137 → / → AMA23_03138*	tRNA-Ala/IS3 family transposase ISAba18
3,242,892	+GTAAG CCAATCT AACGCT	1	1	coding (282/351 nt)	*AMA23_03145 ←*	IS30 family transposase ISAba125

Variant calling was performed using breseq and gdtools software (Parameter: Default)
Reference sequence: A. baumannii AMA23_1 without beta-lactam inhibitor

Table S6: Comparison of the level of expression of genes obtained by RT-qPCR in the AMA22 and AMA23 variants compared to the corresponding wild-type strains.

Gene name	AMA22_1R (Log_2FC)	AMA23_4R (4µg/ml) (Log_2FC)	AMA23_8R (8µg/ml) (Log_2FC)
Outer membrane proteins			
ompA	-3.56 (*)	-4.51	-2.82
carO	-2.79 (*)	-2.59	-2.23
TonB-dependent receptors (TBDRs) and iron homeostasis			
pirA	-2.41 (*)	-0.29	-0.65
bauA	2.67 (*)	0.53	-1.27
piuA	1.21 (*)	0.29	3.06 (*)
ppiA	-0.78 (*)	-0.11	-0.08
Two-component system			
baeS	0.65 (*)	0.10	1.32 (*)
baeR	0.73 (*)	-0.28	-0.87
β-lactamase-encoding genes			
bla_{ADC}	-2.78 (*)	-0.20	0.25
bla_{NDM-1}	-1.68 (*)	1.14 (*)	-1.59
Others			
pbp3	-1.95 (*)	-0.30	-0.80
Biofilms			
bfmS	ND	0.86	0.59
bfmR	ND	3.16 (*)	2.55 (*)

$Log_2FC > 1$ (dark green)

$Log_2FC < -1$ (red)

Log_2FC (0-1) (light green)

Log_2FC (-1-0) (light orange)

ND: not determined

(*) $p < 0.05$
